# A Vascularized, Adipose-Containing Human Skin Equivalent Enables Long-Term Culture and Models Orthopoxvirus-Mediated Immune Suppression

**DOI:** 10.3390/jfb17080374

**Published:** 2026-08-01

**Authors:** Catalina Gaviria Agudelo, Lalitha M. Karchalla, Zachary D. Chandler, Patrick M. McNutt

**Affiliations:** 1 Wake Forest Institute for Regenerative Medicine, Wake Forest University School of Medicine, Winston-Salem, NC 27101, USA; catalina.gaviria@advocatehealth.org (C.G.A.); lalitha.karchalla@wfusm.edu (L.M.K.); zachary.chandler@advocatehealth.org (Z.D.C.); 2 Department of Microbiology and Immunology, Wake Forest University School of Medicine, Winston-Salem, NC 27101, USA

**Keywords:** human skin equivalent, preadipocytes, endothelial cells, optical coherence tomography, disease modeling, cowpox

## Abstract

Human skin is a complex organ whose functions depend on coordinated interactions between the epidermal and stromal layers. Reproducing this architecture in vitro remains challenging, as existing human models reproduce a restricted subset of the structural and functional features inherent to native tissue. Here, we describe a fibrin-based, multicellular human skin equivalent (HSE) composed of primary human keratinocytes, fibroblasts, preadipocytes, and endothelial cells organized into epidermal and stromal compartments. The resulting constructs achieved mature epidermal stratification with appropriate phenotypic markers, developed robust barrier properties, underwent spontaneous endothelial network assembly, and exhibited transcriptional profiles consistent with native skin. Optimized culture conditions supported long-term structural and functional stability through 42 days, maintaining a proliferative basal cell layer and intact epidermal architecture. Non-destructive optical coherence tomography enabled longitudinal monitoring of epidermal growth, providing a practical method for real-time quality assessment. To evaluate their utility for disease modeling, HSEs were challenged with cowpox virus to model infection by a classic dermotropic virus. Infected HSEs reproduced classic epithelial pathologies of human orthopoxvirus infection and exhibited dose-dependent suppression of host interferon signaling pathways, recapitulating known viral immune-evasion mechanisms. Together, these findings establish the vascularized, adipose-integrated HSE as a platform for long-term studies of human skin biology, host–pathogen dynamics, and therapeutic development.

## 1. Introduction

Human skin is a multilayered organ whose functions arise from tightly coordinated interactions among epidermal, dermal, adipose, and vascular compartments. Despite the growing need for advanced human skin models in biomedical research, accurately reproducing the structural and functional complexity of human skin remains challenging [1]. Animal models differ significantly from human skin in anatomy, immune composition, barrier properties, and molecular responses [2,3,4,5,6], while conventional two-dimensional cultures lack the spatial and stromal context required to mimic tissue-level behavior. These limitations restrict the translational utility of animal models and simple cultures for preclinical dermatological studies.

To overcome these limitations, a variety of engineered three-dimensional (3D) skin models have been developed, ranging from epidermal-only constructs [7,8] to full-thickness human skin equivalents (HSEs) containing both epidermal and dermal components [9,10]. However, most current platforms are constrained by short viability, largely due to accelerated terminal differentiation resulting from loss of a proliferative basal layer. In addition, hydrogel contraction driven by fibroblasts further compromises stability, contributing to the structural loss reported across existing models [11,12,13,14,15]. While commercially available models are widely used for standardized toxicology testing, these systems typically maintain experimental viability for only 1–2 weeks [16,17,18,19]. In comparison, extended culture systems capable of reaching 7–8 weeks often lack robust evidence of proper epidermal marker localization or sustained basal cell proliferation [12,14,15]. Moreover, these constructs remain compositionally simple, containing only keratinocytes and fibroblasts, and therefore fail to recapitulate the complex multicellular interactions that regulate skin homeostasis, injury responses, angiogenesis, inflammation, and tissue repair.

Recent studies have highlighted the essential role of epidermal–stromal signaling in maintaining skin integrity [20,21]. Dermal adipocytes, for example, contribute to skin architecture, metabolism, inflammation, wound healing, and epithelial–stromal communication, while endothelial cells support vascular organization, nutrient exchange, angiogenic signaling, and tissue remodeling [20,21,22,23,24,25,26,27,28,29,30,31]. Incorporating these stromal cell types within a compatible extracellular matrix is important in recapitulating the dermo-epidermal junction (DEJ) that is required for skin structural integrity. While emergent skin models containing adipocytes or endothelial cells have improved phenotypic fidelity, they generally include only one or the other of these dermal cell types [32,33,34,35,36,37,38,39,40]. When multiple stromal cell types are present, these platforms frequently fail to achieve full epidermal differentiation, robust barrier formation, long-term homeostatic stability, or physiological responses to pathogens [28,29,30]. Thus, a critical need remains for human skin models that stably integrate epidermal, stromal, adipose, and vascular features while retaining functional integrity.

Here, we describe the generation of a fibrin-based multicellular HSE composed of primary human keratinocytes, fibroblasts, preadipocytes, and endothelial cells organized into distinct epidermal and stromal compartments. This construct supports the formation of a fully stratified epidermis anchored by a proliferative basal epithelial layer; exhibits spontaneous endothelial network assembly, functional adipose differentiation and fibroblast survival; and achieves functional barrier maturation that remains stable through at least six weeks. We further show that optical coherence tomography enables non-destructive, longitudinal monitoring of epidermal development. Finally, using cowpox virus as a model for high-consequence orthopoxvirus infection, we show that the HSE effectively reproduces classic human epithelial pathologies and exhibits dose-dependent suppression of interferon-associated host response pathways. Together, these findings establish the multicellular HSE as a physiologically relevant platform for long-term studies of human skin biology, host–pathogen interactions, injury responses, and therapeutic development.

## 2. Materials and Methods

### 2.1. Cell Isolation and Culture

Primary human dermal fibroblasts, preadipocytes, and keratinocytes were isolated from discarded foreskin tissue. Upon collection, foreskin tissue was sterilized by a brief rinse with 70% ethanol, followed by three washes in phosphate-buffered saline (PBS; Cytiva, Wilmington, DE, USA) supplemented with penicillin/streptomycin/neomycin (PSN; Gibco, Grand Island, NY, USA), and cut into ~25 mm^2^ pieces using sharp scissors. The studies presented here involve cells isolated from 19 distinct donors.

To isolate preadipocytes, the hypodermis was mechanically separated from the skin, finely minced, and incubated in Preadipocyte Growth Medium (PGM) consisting of low-glucose Dulbecco’s Modified Eagle Medium (DMEM; Cytiva, Marlborough, MA, USA), 10% fetal bovine serum (FBS; Gibco) and PSN (Gibco). Preadipocytes migrated from tissue explants within 3–4 days and reached confluence after approximately 2 weeks.

Keratinocytes were isolated by incubating ~25 mm^2^ pieces of skin containing both epidermis and dermis in 2.4 U/mL dispase II (Gibco) in low-glucose DMEM overnight at 37 °C. The next day, the epidermis was carefully peeled from the dermis and dissociated with a 0.12% trypsin/EDTA solution (Gibco) at 37 °C for 30 min. The dissociated epidermis was filtered through a 70 µm cell strainer (Corning, Glendale, AZ, USA), centrifuged at 400× *g* for 5 min, and resuspended in Keratinocyte Growth Medium 2 (KGM2; PromoCell, Heidelberg, Germany) containing SupplementMix (PromoCell; complete KGM2) and supplemented with 10 µM Y-27632 (StemCell, Cambridge, MA, USA) and PSN.

Fibroblasts were isolated by mincing the remaining dermis into small pieces and culturing them in Fibroblast Growth Medium (FGM) composed of high-glucose DMEM (Cytiva), 10% FBS (Gibco) and PSN (Gibco). Fibroblasts began to migrate from the tissue fragments within 3–4 days and reached confluence within 2 weeks, depending on the initial tissue size. Human umbilical vein endothelial cells (HUVECs; Gibco) were purchased from Gibco and maintained according to the manufacturer’s recommendations in Endothelial Cell Growth Medium-2 with supplements (EGM-2; Lonza, Morrisville, NC, USA). For optimal attachment, flasks were precoated with a 0.2% gelatin solution (ScienCell, Carlsbad, CA, USA) prior to cell seeding.

### 2.2. Generation of Human Skin Equivalents (HSE)

To maintain phenotypic identity, keratinocytes, fibroblasts and preadipocytes were used prior to passage eight after isolation, and HUVECs were used before passage six. To generate the subepidermal compartment of the human skin equivalents (HSEs), primary fibroblasts, preadipocytes, and HUVECs were detached using TrypLE (Gibco) and counted, and a density of 1.67 × 10^5^ cells/mL of each cell type was added to a fibrin gel solution consisting of 5 mg/mL fibrinogen (Sigma-Aldrich, St. Louis, MO, USA), 0.25 mM calcium chloride (Sigma-Aldrich), and PBS. Polymerization was initiated by adding 0.2 U/mL thrombin (Sigma-Aldrich) to the fibrinogen cell suspension. The mixture was gently agitated, and 300 µL was transferred to a 24-well cell culture insert (MilliporeSigma, Billerica, MA, USA). Inserts were incubated for 15 min at room temperature, followed by 15 min at 37 °C in a humidified tissue culture incubator with 5% CO_2_ to complete gelation. The resulting gels were submerged overnight in a 1:1:1 mixture of FGM, PGM and EGM-2.

The following day (study day 1), primary keratinocytes were trypsinized and resuspended at a concentration of 1 × 10^6^ cells/mL in complete KGM2 supplemented with 10 µM Y-27632. A total of 1 × 10^5^ keratinocytes were seeded onto each fibrin gel and incubated without media for 1.5 h at 37 °C in 5% CO_2_ to facilitate cell attachment. From 1 to 5 days, the apical and basolateral compartments were maintained in their own specific media: the apical side was supplied with a 1:1 mixture of complete KGM2 and complete Keratinocyte Serum-Free Medium (KSFM; Gibco), the latter consisting of KSFM supplemented with 50 µg/mL bovine pituitary extract and 5 ng/mL epidermal growth factor (Gibco). This apical medium was further supplemented with 10 µM Y-27632 and 8 µg/mL aprotinin (MP Biomedicals, Santa Ana, CA, USA). The basolateral side was maintained in HSE medium, consisting of a 2:2:2:1:1 mixture of FGM, PGM, EGM-2, complete KGM2, and complete KSFM, supplemented to a total of 10 µM Y-27632 and 8 µg/mL aprotinin. The aprotinin was added to prevent fibrin degradation by plasminogen activators secreted by fibroblasts and endothelial cells [41].

At day 5 after keratinocyte plating, both the apical and basolateral compartments were supplied with HSE medium. Once keratinocytes reached full confluency (typically 8 days after seeding), HSEs were transferred to 6-well plates and exposed to the air–liquid interface (ALI) to promote epidermal stratification. At this point, Y-27632 was removed, and the HSE medium was supplemented with 50 µg/mL 2-O-α-D-glucopyranosyl-L-ascorbic acid (vitamin C; Thermo Fisher Scientific, Waltham, MA, USA). At day 15, Y-27632 was reintroduced, and constructs were subsequently maintained in HSE medium containing 10 µM Y-27632 and 50 µg/mL vitamin C, with basolateral media changes performed every other day.

### 2.3. Optical Coherence Tomography (OCT) Imaging

Live HSEs were imaged using the iVivo Ophthalmic OCT system (OcuScience, Henderson, NV, USA) to monitor epidermal development over time. Constructs were placed on a platform under the OCT lens arm, which allowed distance adjustments of the imaging beam relative to the HSE surface. For each image, 30 cross-sectional images were acquired and averaged. Raw OCT data were processed in ImageJ software (version 1.54f) to reduce background noise and enhance image clarity. Epidermal thickness measurements were performed using the Layer Analysis Tool (OcuScience v1.0).

### 2.4. Preadipocyte and Fibroblast Staining

Preadipocytes and fibroblasts were harvested using TrypLE and stained using PKH67 and PKH26 fluorescent cell linker kits (Sigma), respectively. Staining was performed according to the manufacturer’s protocol with minor modifications. Briefly, cells were washed with FBS-free low-glucose DMEM (Cytiva) and resuspended in Diluent C at 4.5 × 10^5^ cells/mL. PKH dyes were diluted to a final concentration of 5 × 10^−5^ M in Diluent C, and equal volumes of the cell suspension were added to the respective dye solutions. After incubating for 5 min at room temperature, an equal volume of FBS (Gibco) was added to stop the reaction. The stained cells were then washed twice with low-glucose DMEM (Cytiva) supplemented with 10% FBS (Gibco), resuspended in 100 µL of FBS (Gibco), and immediately used for HSE assembly.

### 2.5. Whole-Mount Staining

HSEs were fixed in 4% paraformaldehyde overnight and removed from the transwell chambers using a sharp blade. HSEs were transferred to 24-well plates and washed three times with PBS for 1 h. For vascular characterization, HSEs were permeabilized with 0.1% Triton X-100 for 1 h and blocked overnight at 4 °C in 5% goat serum. The following day, HSEs were incubated with rabbit anti-CD31 antibody (Thermo Fisher Scientific PA5-16301; 1:50) at 4 °C for 3 days. After incubation, HSEs were washed three times with PBS for 20 min each and incubated with Alexa Fluor 555 goat anti-rabbit secondary antibody (1:400; Invitrogen, Carlsbad, CA, USA) for 2-3 days at 4 °C. Following another round of three PBS washes for 20 min each, nuclei were counterstained with DAPI (Thermo Fisher Scientific) overnight. For visualization of fluorescently labeled preadipocytes and fibroblasts, no additional staining was performed. For high-resolution microscopy, HSEs were placed in 8-well chamber slides (Ibidi, Fitchburg, WI, USA) and imaged with an ImageXpress Confocal HT.ai High-Content Imaging System (Molecular Devices, San Jose, CA, USA).

### 2.6. Assessment of Epidermal Barrier Integrity

The barrier integrity of HSEs was assessed by evaluating the penetration of the fluorescent dye Lucifer Yellow (Invitrogen). HSEs were rinsed three times with PBS, the basolateral medium was replaced with phenol-free DMEM (Gibco), and 200 µL of 1 mg/mL Lucifer Yellow in phenol-free DMEM was topically applied to the apical surface for 2 h at 37 °C. Control samples were treated with phenol-free DMEM alone. After incubation, the basolateral medium was collected for quantification, and HSEs were rinsed three times with PBS, fixed in 4% paraformaldehyde, and embedded in paraffin. To visualize dye penetration, 5 µm sections were deparaffinized and counterstained with DAPI. Fluorescence imaging was performed using SLIDEVIEW^TM^ VS200 Imaging Scanner (Evident Scientific, Waltham, MA, USA). In parallel, the collected basolateral medium samples were transferred to a 96-well plate along with controls containing phenol-free DMEM (blank) and 1 mg/mL Lucifer Yellow solution. Fluorescence was measured using the SpectraMax i3x spectrofluorometer (Molecular Devices) with an excitation wavelength of 485 nm and emission at 535 nm. The percentage of permeability was calculated as follows: % Permeability = ((Sample − blank)/(Lucifer Yellow solution − blank)) × 100.

### 2.7. Cowpox Infection

HSEs were infected with a recombinant Brighton Red strain of cowpox virus expressing enhanced green fluorescent protein (GFP-cowpox; NR-9597), obtained from the Biodefense and Emerging Infections Resources (Manassas, VA, USA) under the oversight of the National Institute of Allergy and Infectious Diseases of the National Institutes of Health. Viral titer was determined by fluorescent focus assay in Vero E6 cells (ATCC, Manassas, VA, USA; CRL-1586) plated in 96-well dishes (Corning) and maintained in pre-warmed high-glucose DMEM (Cytiva) with 10% FBS (Gibco) and 1× PSN (Gibco). Confluent cell layers were washed with pre-warmed PBS, the medium was replaced with DMEM containing 2% FBS, and the cells were inoculated with 100 μL of ten-fold serial virus dilutions (10^−2^ to 10^−8^) prepared in DMEM/2% FBS. Following a 1 h adsorption window, the inoculum was replaced with 200 μL DMEM/2% FBS for 23 h. Cells were then fixed in 4% paraformaldehyde for 1 h, permeabilized with 0.1% Triton X-100 in PBS, and blocked with 10% BSA (Gibco). Viral protein expression was visualized using rabbit anti-GFP (A11122, 1:400; Invitrogen) followed by Alexa Fluor 555-conjugated goat anti-rabbit secondary antibody (A21428, 1:400; Invitrogen), as described below. Cells were washed with PBS, incubated with 1 μg/mL DAPI (Thermo Fisher Scientific) for 15 min, washed thrice with PBS, and maintained in PBS for imaging. 

Fluorescent images were collected using the ImageXpress Confocal HT.ai High-Content Imaging System (Molecular Devices), and the number of GFP^+^ cells was quantified using the MetaXpress software (version 6.7.2.290, Molecular Devices). The estimated titer was then determined in plaque-forming units (PFU)/mL as the average number of positive cells × 10 divided by the dilution factor. In pilot studies, cells stained with a widely used pan-orthopoxvirus anti-A27L antibody (sc-58210, 1:75; Santa Cruz Biotechnology, Dallas, TX, USA) did not show improved sensitivity versus the anti-GFP antibody. We subsequently used the anti-GFP antibody to quantify viral protein expression. 

For infection studies of keratinocyte monocultures, primary keratinocytes in 96-well plates were infected with GFP-cowpox at 3.8 × 10^3^ PFU/well (nominal MOI = 0.3; *n* = 6 wells per time point). Cells were fixed at 24, 48 or 72 h post-infection (hpi) and counterstained with 1 μg/mL DAPI in PBS. GFP^+^ cells and DAPI^+^ nuclei were enumerated using the MetaXpress software (Molecular Devices) and the mean percentage of infected cells was determined at each time point.

For HSE infections, 18-day-old constructs were challenged either by apical administration of 7.5 × 10^5^ PFU of GFP-cowpox or injection of GFP-cowpox (from 0.1 × 10^4^ to 7.5 × 10^5^ total PFU per HSE) using a 24-gauge needle, with the inoculum distributed across five injection sites (one central and four peripheral). Each site received 5 µL of viral suspension in complete HSE medium. In select experiments, the virus was UV-inactivated by 2 min irradiation at 6500 ± 500 µW/cm^2^ using the Spectrolinker XL-1000 (Spectro-UV, Farmingdale, NY, USA) prior to challenge. Successful inactivation was confirmed by fluorescent focus assay in Vero E6 cells, as described above. Mock-infected controls were injected with complete HSE medium and treated under identical conditions. Following inoculation, constructs were incubated at 37 °C with 5% CO_2_ for 96 h. HSEs were snap-frozen at −80 °C for RNA extraction or fixed in 4% paraformaldehyde for histological and immunohistochemical analyses.

### 2.8. Histology and Immunohistochemistry

HSEs were fixed in 4% paraformaldehyde for 24 h, followed by either cryopreservation or paraffin embedding. For cryopreservation, HSEs were carefully removed from the transwell inserts, transferred to 6-well plates, and washed three times with PBS over the course of 1 h. HSEs were cryoprotected by sequential incubation in 10% sucrose for 1 h, 20% sucrose for 1 h, and 30% sucrose until HSEs sank. Excess cryopreservation solution was removed, and HSEs were placed in disposable embedding molds (Epredia, Kalamazoo, MI, USA) containing FSC 22 frozen section media (Leica Biosystems, Deer Park, IL, USA). Molds were placed on the surface of liquid nitrogen until completely frozen and stored at −80 °C. Frozen tissue sections (14 µm) were cut with a Leica cryotome and baked at 60 °C for 45 min to ensure tissue adhesion. Sections were stained with hematoxylin and eosin (H&E) for morphological evaluation.

For paraffin embedding, fixed HSEs were transferred to 70% ethanol and processed to obtain paraffin blocks. Tissue sections (5 µm) were obtained using a Leica microtome and baked at 60 °C for 1 h to ensure tissue adhesion. Sections were deparaffinized and stained with H&E for morphological evaluation. Brightfield images were captured using a SLIDEVIEW^TM^ VS200 Imaging Scanner (Evident Scientific). Epidermal thickness was measured using OlyVIA software (version 4.1, Evident Scientific).

For immunohistochemistry, 5 µm paraffin sections were deparaffinized and subjected to antigen retrieval using either 10 mM citrate buffer with 0.05% Tween-20 (pH 6) or 10 mM Tris-EDTA buffer with 1 mM EDTA and 0.05% Tween-20 (pH 9). Antigen retrieval was conducted at pH 6 for antibodies against cytokeratin 5, CD31, p63, Ki67 and GFP; and at pH 9 for all other antibodies. Both paraffin and frozen sections were permeabilized with 0.1% Triton X-100 for 5 min and then blocked at room temperature using Protein Block Solution (Agilent Dako, Santa Clara, CA, USA).

The following primary antibodies were diluted in Protein Block Solution and applied overnight at 4 °C: rabbit anti-cytokeratin 5 (ab64081, 1:120; Abcam, Waltham, MA, USA), rabbit anti-cytokeratin 10 (ab76318, 1:6000; Abcam), Alexa Fluor 647 rabbit anti-cytokeratin 10 (ab194231, 1:100; Abcam), rabbit anti-loricrin (ab198994, 1:250; Abcam), rabbit anti-claudin-1 (ab211737, 1:250; Abcam), Alexa Fluor 488 anti-vimentin (ab185030, 1:400; Abcam), rabbit anti-CD31 (PA5-32321, 1:110; Thermo Fisher Scientific), mouse anti-p63 (CM 163A, 1:300; Biocare Medical, Pacheco, CA, USA), rabbit anti-Ki67 (ab243878, 1:100; Abcam) and rabbit anti-GFP (A11122, 1:400; Invitrogen). Following primary antibody incubation, sections were washed with PBS and incubated for 1 h at room temperature with Alexa Fluor 555-conjugated goat anti-rabbit secondary antibody (A21428, 1:400; Invitrogen) or Alexa Fluor 488-conjugated goat anti-mouse secondary antibody (A11001, 1:400; Invitrogen) diluted in Protein Block Solution. After additional PBS washes, nuclei were counterstained with DAPI. Slides were mounted using ProLong^TM^ Glass Antifade Mountant (Thermo Fisher Scientific), and images were captured using the SLIDEVIEW^TM^ VS200 (Evident Scientific) or the BZ-X800 (Keyence).

### 2.9. Oil Red O Staining

Cryosections were rinsed in deionized water and placed in absolute propylene glycol for 5 min. Sections were then stained with Oil Red O solution (Sigma) for 10 min at 60 °C, followed by a 5 min incubation in 85% propylene glycol. Mayer’s hematoxylin was applied for 3 min, and slides were thoroughly washed under running tap water for 2 min. Samples were mounted using Fluoromount (Thermo Fisher Scientific), and brightfield images were acquired using a BZ-X800 microscope (Keyence).

### 2.10. Quantification of Basal Cell Density and Proliferation Index

Basal cell density was quantified as the number of p63-positive cells per 100 μm of basal epidermal length, as measured using the Keyence BZ-X800 analyzer software. The proliferation index was calculated as the percentage of Ki67-positive cells relative to the total number of p63-positive cells within the basal layer of the HSEs. For both measurements, at least three fields of view were quantified per HSE. The total number of HSEs is described in each figure legend.

### 2.11. RNA Extraction

Total RNA was extracted from HSEs using the RNeasy Midi Kit (QIAGEN, Germantown, MD, USA) according to the manufacturer’s instructions. Briefly, constructs were removed from transwell inserts, snap-frozen and stored at −80 °C until processing. Frozen tissues were homogenized in RLT lysis buffer using a Fisherbrand^TM^ 850 Homogenizer (Thermo Fisher Scientific). The lysates were then processed through the RNeasy Midi spin columns, including on-column DNase treatment to remove genomic DNA contamination. RNA was eluted in 150 µL of RNase-free water, quantified using a NanoDrop spectrophotometer (Thermo Fisher Scientific), and analyzed by the Agilent 2100 Bioanalyzer (Agilent Technologies, Santa Clara, CA, USA). Samples with RNA integrity numbers (RIN) exceeding 8.0 were retained for downstream analyses.

### 2.12. RNA Sequencing and Analysis

Total RNA from each sample was submitted to Novogene (Sacramento, CA, USA) for poly(A)-selected mRNA sequencing. Libraries were sequenced on a NovaSeq 6000 using a 2 × 150 bp paired-end configuration, generating a minimum of 30 million read pairs per sample. Raw sequencing-read quality was assessed using FastQC version 0.12.1. Adapter sequences and low-quality bases were removed using Cutadapt version 5.1 [42]. High-quality reads were aligned to the human reference genome GRCh38/hg38 using STAR version 2.7.11b [43]. Gene-level counts were generated using featureCounts version 2.1.1 [44] with the Ensembl release 115 annotation. Read counts were processed using edgeR version 4.2.2 [45]. To account for differences in library composition and sequencing depth, normalization factors were calculated using the trimmed mean of M-values method. TMM-normalized log2 counts per million were calculated for visualization and descriptive comparison of gene expression patterns. To identify tissue- and cell-type-specific expression profiles associated with our differentially expressed genes (DEGs), we performed a functional enrichment analysis using the Database for Annotation, Visualization, and Integrated Discovery (DAVID 2021) [46]. Genes were ranked by their mean TMM-normalized counts per million across all samples, and the 1000 most abundant genes were mapped against the UP_TISSUE categories. Statistical significance of the over-represented cell types and tissues was determined using a modified Fisher’s exact test (*p*-value < 0.05), and *p*-values were adjusted for multiple testing using the Benjamini–Hochberg false discovery rate (FDR) correction.

### 2.13. NanoString Gene Expression Analysis

The NanoString nCounter^®^ Analysis System (Bruker Spatial Biology, Bothell WA, USA) was used to assess gene expression related to host responses to infection. The nCounter^®^ Host Response Panel (XT-HHR-12), which targets 785 genes, including 12 internal reference genes, was utilized. This panel covers key components of the host response, including susceptibility, interferon signaling, innate immune activation, and tissue homeostasis. A total of 150 ng of RNA per sample was used for hybridization, which was performed according to the manufacturer’s protocol.

### 2.14. Statistics and Data Analysis

Statistical analyses were performed using GraphPad Prism (version 10.4.1, GraphPad Software, Boston, MA, USA). One-way analysis of variance (ANOVA) was used for multiple-group comparisons, and differences were considered statistically significant at *p* ≤ 0.05. All graphs were generated using GraphPad Prism. NanoString data were analyzed using the Advanced Analysis module of the nSolver^TM^ Analysis Software (version 4.0, Bruker Spatial Biology). Genes were considered differentially expressed with log_2_(fold change) ≤ −1 or ≥1 and an FDR ≤ 0.05, and the results were visualized as volcano plots. To identify significantly perturbed biological pathways following infection, Ingenuity Pathway Analysis (IPA) software (version 159584291, QIAGEN) was used. A list of genes included in the nCounter^®^ Host Response Panel, along with their corresponding log_2_(fold change) and *p*-values, was uploaded to IPA. Canonical pathways were evaluated using the right-tailed Fisher’s exact test, and significantly enriched pathways were visualized as bar charts. Statistical tests and n-values are provided in the corresponding figure legends.

## 3. Results

### 3.1. Engineering a Multicellular Human Skin Equivalent with a Fully Stratified Epidermis

**Figure 1:**
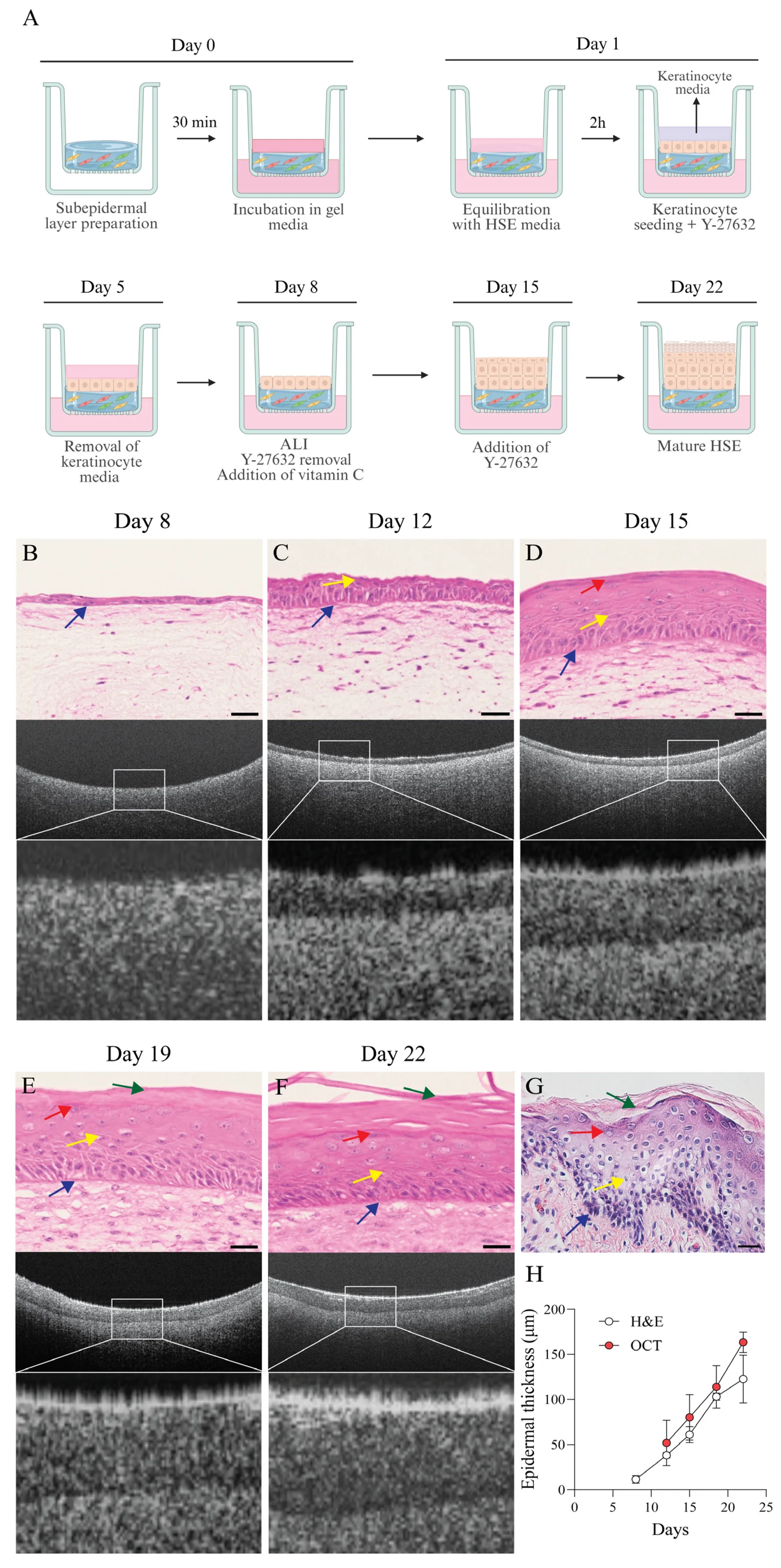
Generation and morphological characterization of HSEs. (**A**) Schematic workflow for the generation of HSEs. (**B**–**F**) Upper panels: H&E images of HSEs from day 8 to day 22. Arrows indicate epidermal strata (blue: basal layer; yellow: spinous layer; red: granular layer; green: stratum corneum). Middle and lower panels: corresponding OCT images. Lower panels: higher-magnification views of marked OCT regions. (**G**) H&E image of native human skin. (**H**) Mean ± SEM epidermal thickness in both OCT and H&E images (*n* = 5–14 HSEs). Scale bars = 25 µm.

A sequential assembly strategy was used to generate multicellular human skin equivalents (HSEs) containing major cellular components of the epidermal and dermal compartments (Figure 1A). The stromal compartment was formed by polymerizing a fibrin-based hydrogel containing primary human fibroblasts, preadipocytes, and endothelial cells on a transwell membrane. Fibrin was selected because its mechanical properties, degradation and contraction, and porosity can be controlled while supporting cell growth, migration, nutrient diffusion, and tissue remodeling [47,48,49]. Basal keratinocytes were subsequently seeded onto the polymerized stromal compartment. The nascent HSEs were maintained under submerged conditions for 8 days to support keratinocyte expansion and were then transitioned to air–liquid interface (ALI) culture to initiate epidermal differentiation.

In pilot studies, submerging the apical surface in HSE medium prior to transition to ALI resulted in hyperdifferentiation, involving the loss of basal and granular layers and the formation of a hyperkeratotic stratum corneum (Supplemental Figure S1). To promote epidermal stratification while limiting premature terminal differentiation, we evaluated a range of culture medium formulations and assessed epithelial morphology on day 15. We eventually identified a three-step culture protocol, which produced the most physiologically organized epidermal morphology among the conditions evaluated. Before transition to ALI, the apical compartment was maintained in a 1:1 mixture of complete KGM2 and complete KSFM supplemented with 10 µM Y-27632, a Rho-associated protein kinase inhibitor [50]. Following the transition to ALI, Y-27632 was removed from the basolateral medium, and 50 µg/mL vitamin C was added to support epidermal stratification and barrier formation [51,52]. Y-27632 was reintroduced into the basolateral medium on day 15 with the aim of limiting excessive terminal differentiation and maintaining the basal keratinocyte population. Under these optimized conditions, HSEs developed a well-stratified epidermis containing distinct basal, spinous, granular, and cornified layers characteristic of native human epidermal architecture (Figure 1B–G).

To characterize epidermal development longitudinally, optical coherence tomography (OCT) was used to assess HSE morphology non-destructively over time (Figure 1B–F, middle and lower panels). OCT-derived measurements of epidermal thickness showed a temporal pattern similar to that obtained from histological sections (Figure 1H), supporting the use of OCT for longitudinal monitoring of epidermal development without destructive tissue collection.

### 3.2. HSEs Recapitulate Key Phenotypic, Molecular, and Barrier Features of Native Human Skin

**Figure 2:**
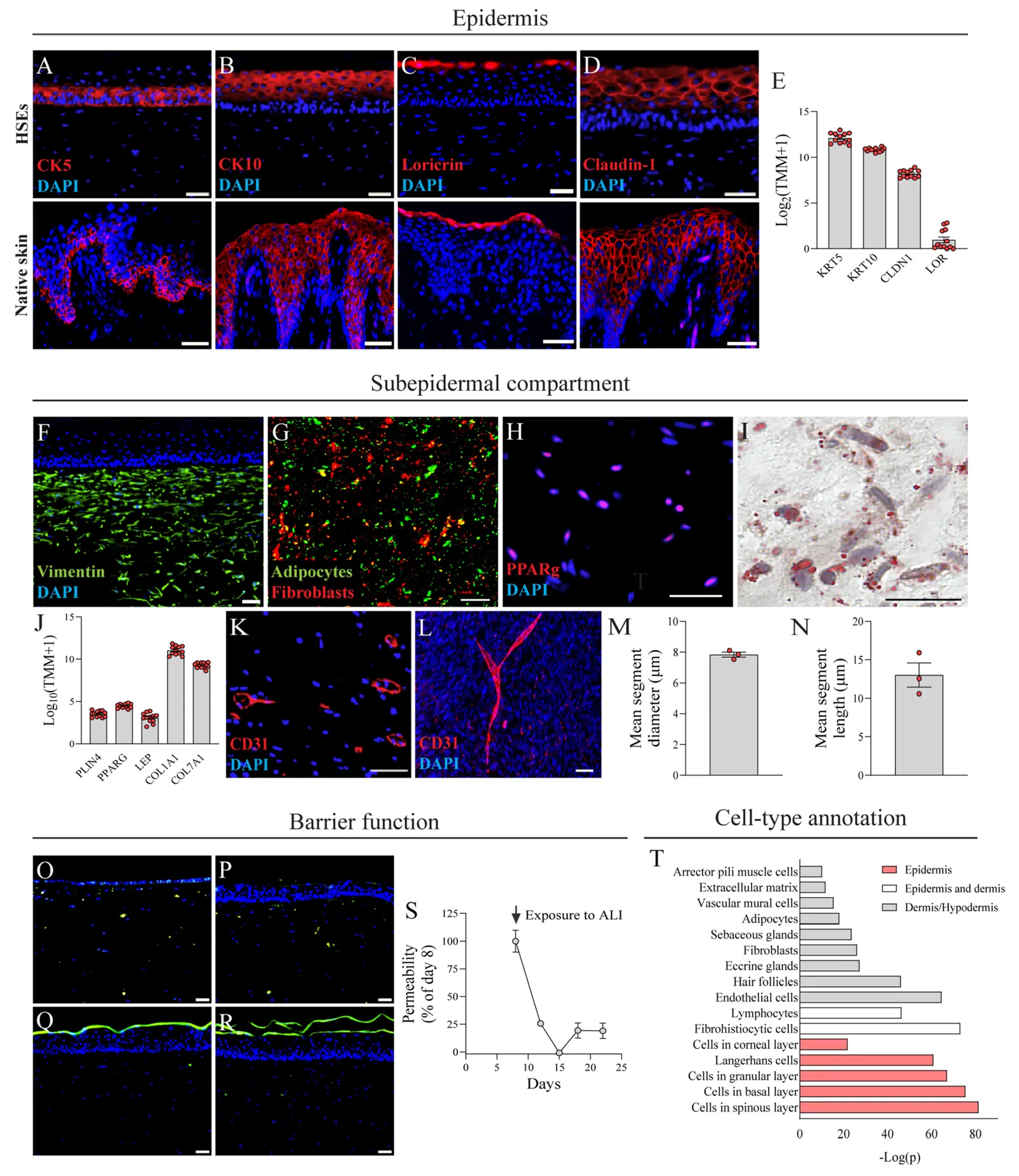
Phenotypic and transcriptional characterization of HSEs. (**A**–**D**) IHC of HSEs (upper panels) and native human skin (lower panels) for (**A**) cytokeratin 5, (**B**) cytokeratin 10, (**C**) loricrin, and (**D**) claudin-1. (**E**) Mean ± SEM expression levels of epidermal differentiation genes in HSEs (*n* = 12 HSEs). (**F**) Vimentin IHC. (**G**) Maximum-intensity projection showing stromal distribution of preadipose lineage cells (green) and fibroblasts (red). (**H**) PPARγ2 IHC. (**I**) Representative Oil Red O staining showing lipid droplet accumulation. (**J**) Mean ± SEM expression levels of fibroblast-specific and adipose-specific genes in HSEs (*n* = 12 HSEs). (**K**,**L**) CD31 IHC showing (**K**) capillary-like lumens and (**L**) capillary organization. (**M**,**N**) Mean ± SD of (**M**) diameter and (**N**) segment length of capillary-like structures (*n* = 3 HSEs). (**O**–**S**) Lucifer Yellow imaging at (**O**) day 8, (**P**) day 12, (**Q**) day 15, and (**R**) day 22 of culture. (**S**) Mean ± SEM quantitation of Lucifer Yellow penetration to the basolateral medium from days 8 to 22, normalized to day 8 (*n* = 3 HSEs). (**T**) DAVID annotation tool analysis of abundant HSE transcripts (*n* = 6 HSEs). Red: epidermal annotations; gray: dermal annotations; white: shared dermal and epidermal annotations. Scale bar = 25 µm for (**I**) and 50 µm for all other images.

Following structural maturation, HSEs were evaluated for epidermal differentiation, stromal cell identity, vascular organization, and barrier function. At day 22, immunofluorescence studies revealed the localization of cytokeratin 5 (CK5) to the basal epithelium (Figure 2A, upper panel), cytokeratin 10 (CK10) to the suprabasal layers (Figure 2B, upper panel), and loricrin to the upper differentiated epidermis and stratum corneum (Figure 2C, upper panel) [53]. Claudin-1, a tight junction protein that contributes to epidermal barrier integrity, was predominantly associated with the upper spinous and granular layers (Figure 2D, upper panel). These staining patterns are similar to those observed in native human skin (Figure 2A–D, lower panels) and are consistent with the formation of a stratified epidermis. Bulk transcriptomic profiling further detected transcripts associated with basal, spinous, granular, and cornified epidermal differentiation programs (Figure 2E).

We next examined fibroblast, adipose-lineage, and endothelial populations within the subepithelial compartment. Vimentin-positive mesenchymal cells were distributed throughout the stromal layer (Figure 2F). Because vimentin does not distinguish fibroblasts from adipose-lineage cells, fluorescent membrane labels were used to track the two cell populations separately (Figure 2G). PPARγ2 expression, together with intracellular lipid accumulation, provided molecular and phenotypic evidence of adipogenic differentiation (Figure 2H,I) [54,55,56]. Transcriptomic analysis detected adipose-associated genes, including PPARG, PLIN4, and LEP [57,58,59], as well as extracellular-matrix-associated genes, including COL1A1 and COL7A1 [60]. Together with fluorescent cell tracking and histological analyses, these findings supported the sustained viability of fibroblast and adipose-lineage populations as well as the differentiation of adipose cells (Figure 2J).

CD31-positive endothelial cells self-organized into branching, lumen-containing, capillary-like structures throughout the stromal compartment (Figure 2K,L; Supplemental Video S1). A total of 2798 segments imaged across three HSEs exhibited a mean diameter of 7.83 ± 0.16 µm and a mean segment length of 13.03 ± 1.56 µm (Figure 2M,N).

The development of an epidermal permeability barrier was assessed by apical application of the membrane-impermeant fluorescent tracer Lucifer Yellow (Figure 2O–S) [61]. At days 8 and 12, Lucifer Yellow readily penetrated the epidermis and accumulated within the underlying tissue and basolateral compartment, indicating the absence of an epidermal barrier. Lucifer Yellow penetration progressively decreased between days 8 and 18. By day 18, Lucifer Yellow penetration was minimal and remained low through day 22. Destruction of the epithelial barrier by apical addition of 1% SDS increased Lucifer Yellow penetration, confirming that reduced dye penetration reflected development of an epidermal permeability barrier (Supplemental Figure S2).

To assess whether the most highly expressed HSE genes were enriched for skin- and constituent-cell-associated annotations, the 1000 most abundant genes from the bulk transcriptomic data were identified and subjected to tissue-enrichment analysis using the Database for Annotation, Visualization, and Integrated Discovery (DAVID) (Figure 2T). DAVID analysis identified significant overrepresentation of genes annotated as enriched in human skin (FDR = 5.9 × 10^−53^). Additional significantly enriched annotations were associated with epidermal differentiation, fibroblast and adipose biology, endothelial cells, extracellular-matrix organization, and skin-associated epithelial programs. Together with the histological, immunofluorescence, and functional analyses, these enrichment results support the conclusion that HSEs reproduce key phenotypic markers of human skin.

### 3.3. HSEs Maintain Structural and Phenotypic Stability During Long-Term Culture

**Figure 3:**
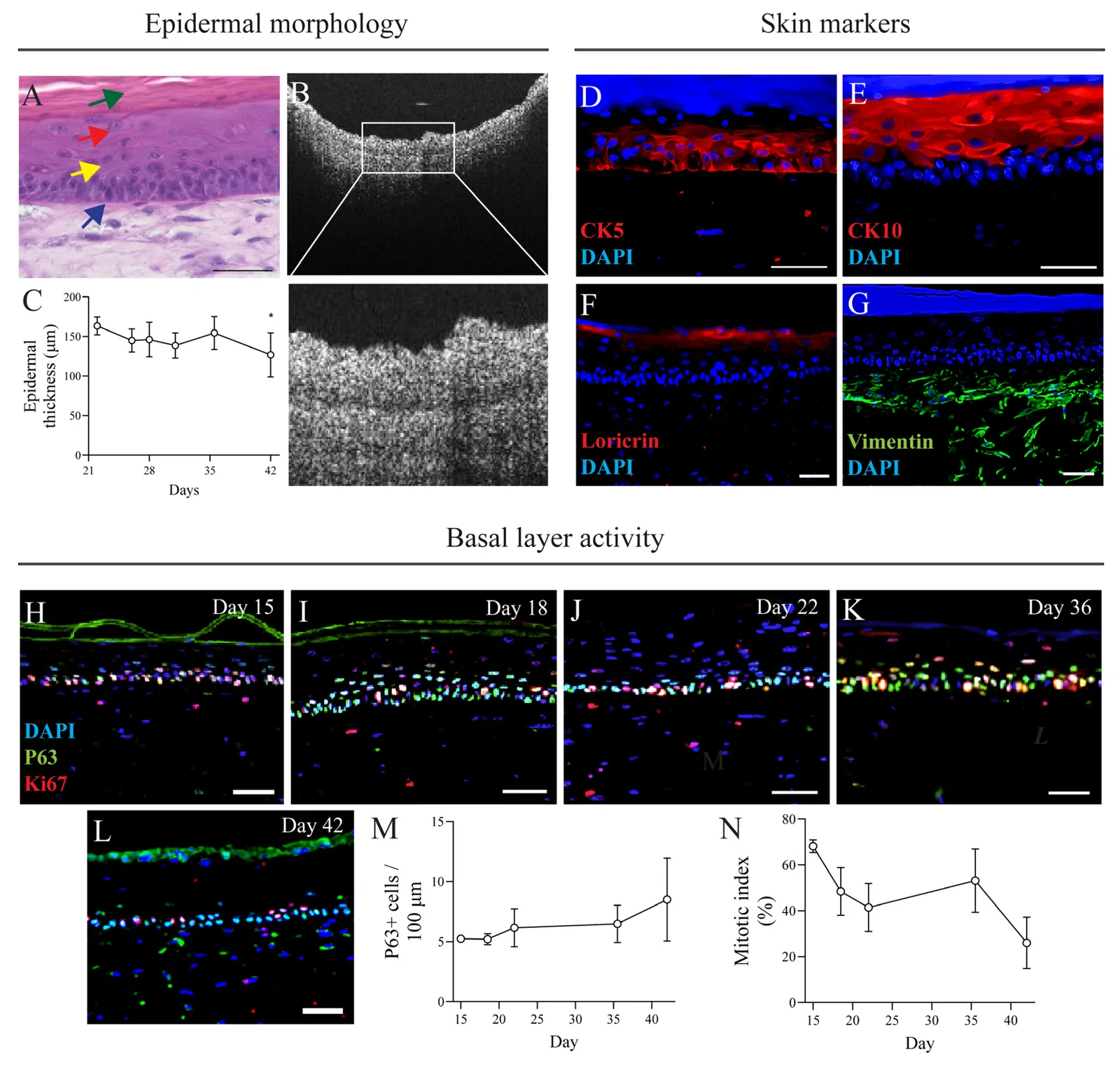
Structural and phenotypic characterization of HSEs during long-term culture. The stability of HSEs after 42 days in culture was evaluated using histological and immunophenotypic analyses. (**A**) H&E staining showing epidermal stratification. Arrows indicate epidermal strata (blue: basal layer; yellow: spinous layer; red: granular layer; green: stratum corneum). (**B**) OCT image illustrating epidermal thickness; the lower panel shows a higher-magnification view of the boxed region. (**C**) Mean ± SD epidermal thickness in OCT images (*n* = 3–7 HSEs). (**D**–**G**) IHC for (**D**) cytokeratin 5, (**E**) cytokeratin 10, (**F**) loricrin and (**G**) vimentin. (**H**–**N**) Basal cell density and proliferation were assessed by p63 and Ki67 immunostaining at (**H**) day 15, (**I**) day 18, (**J**) day 22, (**K**) day 36 and (**L**) day 42. (**M**) Mean ± SD basal cell density, expressed as the number of p63^+^ cells per 100 µm of basal layer (*n* = 3–5 HSEs). (**N**) Mean ± SD proliferation index, calculated as the percentage of Ki67^+^ cells relative to p63^+^ basal cells (*n* = 3–5 HSEs). For statistical analysis, one-way ANOVA with Tukey’s test was applied: * *p* ≤ 0.05. Scale bar = 50 µm.

To evaluate long-term structural and phenotypic stability of HSEs, epidermal morphology and expression of key epidermal and stromal markers were characterized through day 42 (Figure 3). HSEs retained a stratified epidermis with continuous coverage across the construct (Figure 3A,B), and epidermal thickness did not differ significantly over time (Figure 3C). HSEs preserved columnar basal keratinocytes and suprabasal layers throughout 42 days. Thickening of the cornified layer was occasionally observed (Supplemental Figure S3A), which may reflect the absence of mechanical desquamation under in vitro conditions.

Immunophenotypic analysis further supported maintenance of epidermal organization from day 22 through day 42 (Figure 3D–F). Although low-intensity CK5 staining extended into the immediate suprabasal layers at day 42, CK10 (Figure 3E) and loricrin (Figure 3F) retained their expected suprabasal and upper-epidermal distributions, while Ki67 and p63 (Figure 3L) expression was consistent with the maintenance of a stratified epidermis. Stromal cells continued to be distributed throughout the dermal compartment (Figure 3G), while Masson’s trichrome staining demonstrated the production of collagen-rich extracellular matrix within the stroma (Supplemental Figure S3B).

Because maintenance of the basal keratinocyte population is essential for epidermal homeostasis [62], we quantified basal cell density and proliferation from day 15 through day 42. p63-positive basal cells and Ki67-positive proliferating cells were detected at each time point (Figure 3H–L). Basal cell density, measured as the number of p63-positive cells per unit length of basal epidermis, remained relatively stable over time (Figure 3M). The proliferative index, defined as the percentage of p63-positive basal cells that co-expressed Ki67, was also largely maintained, although it was slightly decreased at day 42 (Figure 3N). The preservation of p63-positive basal cell density despite the lower proportion of Ki67-positive basal cells suggests that the basal compartment remained intact while a smaller proportion of cells were actively cycling through 42 days of culture. Together, these findings indicate that HSEs preserve epidermal stratification, a p63-positive basal cell compartment, stromal mesenchymal-cell distribution, and collagen-rich extracellular matrix through 42 days of culture.

### 3.4. HSEs Support Orthopoxvirus Infection and Reveal Dose-Dependent Suppression of Host Interferon Responses

Orthopoxviruses are clinically relevant dermotropic DNA viruses that produce characteristic epithelial pathology and modulate innate antiviral responses, including interferon signaling [63,64,65,66]. We used a GFP-expressing Brighton Red cowpox virus [67] to determine whether HSEs recapitulate orthopoxvirus-associated epithelial pathology and modulation of host antiviral responses [68].

GFP-cowpox titers were determined in Vero E6 cells, which are deficient in type I interferon production and are therefore highly permissive to many viral infections, including orthopoxvirus infection [69]. Before conducting infection studies in HSEs, we assessed whether GFP-cowpox could infect primary human keratinocytes. Keratinocyte monolayers in 96-well plates were inoculated with GFP-cowpox at a nominal multiplicity of infection (MOI) of 0.3 (as determined in Vero cells). GFP expression was measured in uninfected controls and at 24, 48, and 72 h post-infection (hpi). The proportion of GFP-positive cells increased from 0% prior to exposure to 1.1% at 24 hpi, 5.5% at 48 hpi and 15.3% at 72 hpi. The resulting Hill slope was 2.8 (R^2^ = 0.73), consistent with a model in which there is an initial infection of a small proportion of cells followed by viral spread through secondary transmission. Having established that GFP-cowpox could infect and spread within primary keratinocytes, we next evaluated infection of HSEs.

**Figure 4:**
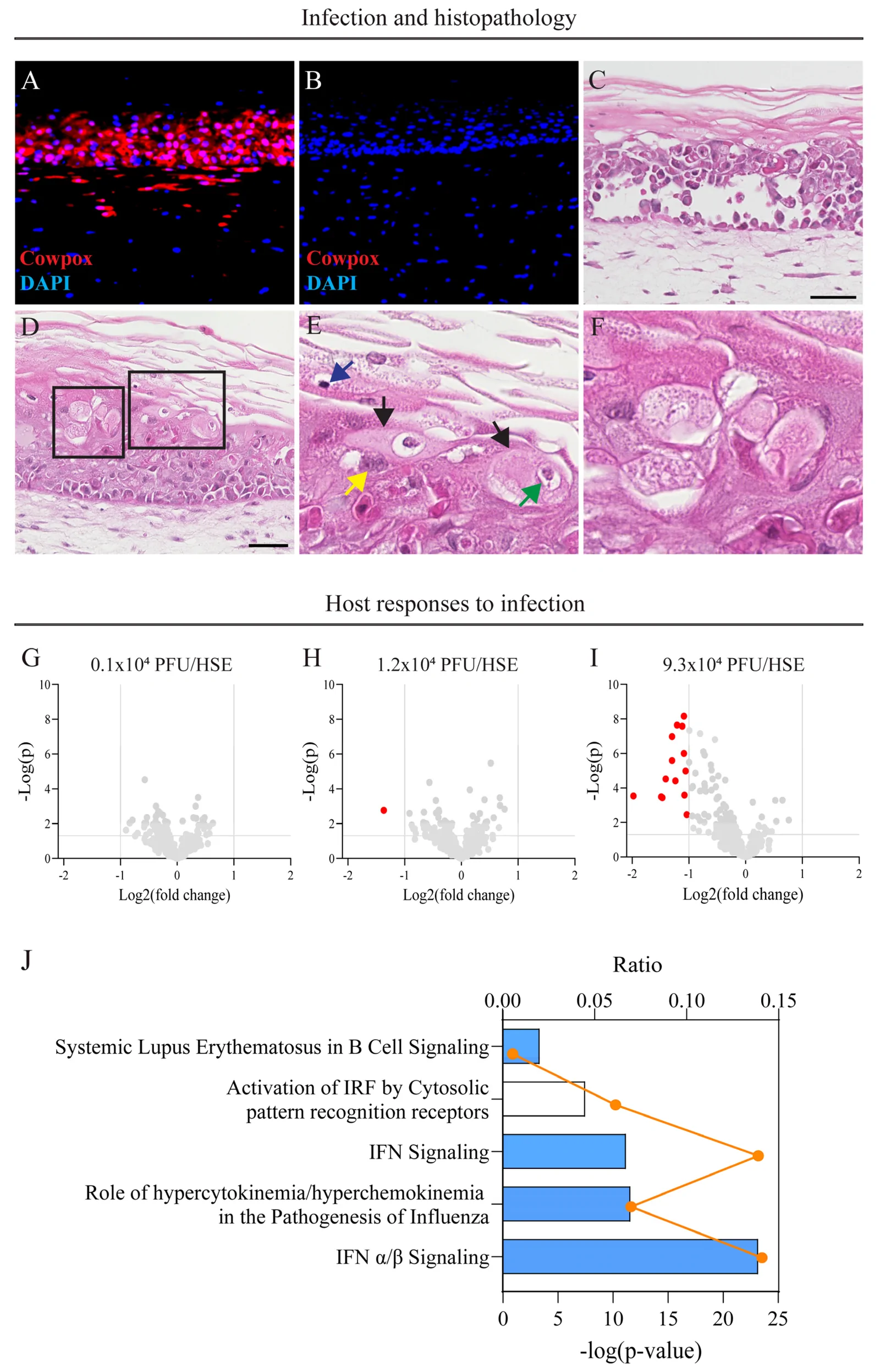
Structural and transcriptional responses of HSEs infected with cowpox. (**A**,**B**) Immunohistochemical staining of HSEs infected with (**A**) 9.3 × 10^4^ PFU GFP-cowpox or (**B**) UV-irradiated virus. (**C**,**D**) H&E staining of HSEs infected with (**C**) 7.5 × 10^45^ PFU showing suprabasal blister formation or (**D**) H&E staining of HSEs infected with 9.3 × 10^4^ PFU showing cytopathic effects. (**E**,**F**) Higher-magnification views illustrate hallmark features of infection, including (**E**) pyknotic nuclei (blue arrow), cell ballooning (black arrows), binucleation (yellow arrow), micronucleus formation (green arrow), and (**F**) nuclear dissolution. (**G**–**I**) Volcano plots displaying differentially expressed genes in infected vs. vehicle-treated HSEs across increasing cowpox doses: (**G**) 0.1 × 10^4^ PFU, (**H**) 1.2 × 10^4^ PFU and (**I**) 9.3 × 10^4^ PFU as quantified by NanoString analysis. Red: significantly downregulated genes; vertical gray lines: Log2 up- or downregulation; and horizontal line: FDR = 0.05 (*n* = 6 HSEs). (**J**) Significantly perturbed canonical pathways following infection with 9.3 × 10^4^ PFU. Blue indicates suppressed pathways. Orange reflects the ratio of affected genes in each pathway. For statistical analysis, Fisher’s exact test was used. −Log(FDR)  >  1.3 was considered significant (*n* = 6 HSEs). Scale bar = 25 µm for (**E**,**F**) and 50 µm for all other images.

We first attempted to infect HSEs by topical addition of 7.5 × 10^5^ PFU GFP-cowpox virus to the apical surface. Consistent with the formation of an impermeable epidermal barrier in HSEs, apical exposure did not produce detectable infection at 96 hpi (Supplemental Figure S4). To bypass the epidermal barrier, GFP-cowpox was injected directly into the subepidermal compartment at total doses ranging from 0.1 × 10^4^ to 7.5 × 10^5^ PFU. Each HSE was injected at five locations, with each location receiving one-fifth of the total dose. Direct injection resulted in successful infection, as measured by GFP expression at 96 hpi. GFP-positive cells were widespread within the epidermis and, to a lesser extent, the stromal compartment (Figure 4A). In contrast, GFP was not detected in HSEs injected with UV-inactivated GFP-cowpox (Figure 4B), confirming that detection of GFP reflects de novo viral gene expression rather than residual GFP associated with the input virus.

Histological analysis of GFP-cowpox-injected HSEs at 96 hpi revealed dose-associated epithelial pathology at inoculation doses exceeding 9.3 × 10^4^ PFU per HSE. Histopathological changes included epidermal disruption and suprabasal vesication, resembling features reported in poxvirus-associated lesions (Figure 4C) [70,71]. Additional cytopathic changes included pyknotic nuclei, cellular ballooning, binucleation, and nuclear dissolution (Figure 4D–F). Micronucleus-like nuclear structures were also observed in infected tissues (Figure 4E, green arrow). These structures may reflect infection-associated nuclear damage or mitotic abnormalities, although their identity and underlying mechanism require further validation. In contrast, ≤1.2 × 10^4^ PFU did not produce overt histopathological changes by 96 h.

Orthopoxviruses employ multiple mechanisms to antagonize host innate antiviral responses [65,66,72]. To determine whether cowpox infection was associated with attenuation of antiviral signaling in HSEs, RNA was collected from infected and control HSEs at 96 hpi and analyzed by fluorescent RNA hybridization using a 773-gene NanoString human host response panel. Increasing GFP-cowpox dose was associated with greater downregulation of type I and type III interferon-related genes relative to uninfected controls (Figure 4G–I). Pathway analysis similarly identified reduced expression of genes associated with interferon signaling, together with decreased expression of selected cytokine- and chemokine-related genes (Figure 4J). Collectively, these data demonstrate that HSEs support cowpox virus infection and recapitulate key orthopoxvirus-associated features of cutaneous disease, including dose-associated epithelial pathology and attenuation of type I and type III interferon-related transcriptional responses.

## 4. Discussion

In this study, we developed a fibrin-based, multicellular human skin equivalent integrating primary keratinocytes, fibroblasts, preadipocytes, and endothelial cells within organized epidermal and stromal compartments. The principal advance of this model is the combination of long-term epidermal maintenance, preservation of a proliferative basal keratinocyte population, adipose-lineage and endothelial stromal features, non-destructive longitudinal imaging, barrier formation, and responsiveness to viral challenge within a single platform. Together, these characteristics support the use of HSEs as a physiologically relevant in vitro system for investigating human skin biology, epidermal–stromal interactions, injury responses, host–pathogen interactions, and the preclinical evaluation of medical countermeasures.

A key feature of the model was the staged culture protocol used to support epidermal maturation and long-term tissue maintenance. Y-27632 is widely used to promote the expansion of primary keratinocytes in an undifferentiated state and is typically removed to permit stratification [73,74], but its effects following the establishment of a fully stratified epidermis remain incompletely characterized. Here, we found that the reintroduction of Y-27632 after stratification was associated with preservation of epidermal architecture and maintenance of p63-positive basal keratinocytes through 42 days of culture. The persistence of Ki67-positive basal cells, together with the appropriate spatial localization of early and terminal differentiation markers, indicated that a population of actively cycling basal keratinocytes was retained without overt disruption of epidermal differentiation or stratification. The staged use of Y-27632 may therefore represent a practical strategy for extending the experimental lifespan of engineered epithelial tissues. The resulting culture stability may facilitate studies of chronic exposures, delayed therapeutic responses, and longer-term tissue-remodeling processes that are difficult to evaluate in shorter-lived HSE systems.

In addition to the establishment of a stratified, long-lasting epithelium, the stromal compartment further distinguishes this model from many existing skin platforms by incorporating primary preadipocytes, fibroblasts and endothelial cells within a fibrin-based matrix. Phenotypic differentiation and organization of these populations were supported by intracellular lipid accumulation and adipose-associated gene expression, the persistence of labeled fibroblast and adipose-lineage populations, and extracellular-matrix-associated gene expression. Endothelial cells spontaneously organized into branching, capillary-like networks, a feature that is less commonly observed in full-thickness engineered skin models without prevascularization or microfluidic approaches [75,76,77]. Although these networks were not perfused, their self-organization demonstrates that the fibrin stromal environment supports endothelial migration and multicellular network formation. The combination of adipose-lineage features, spontaneous endothelial organization, and prolonged epidermal maintenance is markedly different from that of many engineered skin systems [28,38,39] and commercial platforms [9,10]. These stromal components are critically important for physiological skin health [56,57,59,60,78] and support the use of HSEs for disease modeling, wound-healing studies, dermatotoxicology, permeability testing, and therapeutic screening.

Optical coherence tomography provided a non-destructive method for monitoring epidermal development over time. Conventional assessment of epidermal maturation relies largely on histological analysis, which requires tissue fixation and the use of separate constructs at each time point, thereby limiting longitudinal evaluation [79]. In contrast, OCT enabled repeated visualization of HSE morphology throughout culture, and OCT-derived epidermal thickness measurements showed temporal patterns similar to those obtained from histological sections. OCT, therefore, offers a means of monitoring tissue maturation without disrupting the construct or requiring the collection of replicate tissues at every time point. Improvements in imaging resolution and penetration depth may further expand its utility for evaluating full-thickness architecture, barrier-associated changes, tissue injury, and remodeling in engineered skin and other organotypic tissue models.

The response of HSEs to the cowpox virus provided an initial demonstration of the model’s utility for studying cutaneous host–pathogen interactions. Animal models and two-dimensional cell cultures have advanced the understanding of orthopoxvirus biology but do not fully reproduce the architecture of human skin or the complexity of multicellular dermal responses [5,6,80,81]. Most animal models also fail to reproduce the full spectrum of blistering observed in human disease [82], producing microblisters or dermo-epidermal separation rather than the suprabasal and subcorneal vesication characteristic of human lesions [83,84]. Following direct subepidermal inoculation with the cowpox virus, HSEs exhibited several clinical [70,71] and in vitro [85,86] features associated with orthopoxvirus infection, including localized viral spread, pyknosis, cellular ballooning, binucleation, epidermal disruption, and suprabasal vesication. These findings indicate that the spatial organization of the HSE permits the development of tissue-level epithelial pathology that cannot be fully represented in keratinocyte monolayers and is not typically reproduced in animal models.

Micronucleus-like structures were also observed in infected tissues. Nuclear abnormalities of this type have been associated with genotoxic stress, abnormal mitosis, and infectious diseases [87,88,89,90,91]. However, their identity and biological significance remain uncertain. Further evaluation using markers of chromatin organization, nuclear-envelope integrity, mitotic progression, and DNA damage will be required to determine whether these structures represent bona fide micronuclei, nuclear fragmentation, or another form of infection-associated nuclear injury. Furthermore, cowpox infection was associated with dose-related attenuation of type I and type III interferon-related transcriptional responses. This finding is consistent with the established ability of orthopoxviruses to antagonize innate antiviral signaling [65,66]. Similarly, monkeypox-infected skin organoids have been reported to exhibit limited interferon activation [86].

Several additional features of HSEs should be considered when interpreting their translational relevance. First, the current version of HSEs lacks resident and recruited immune cell populations, limiting its ability to reproduce the full range of innate inflammatory immune responses associated with injury and infection. Second, the endothelial compartment remains an area for further development. Although endothelial cells formed lumen-containing, capillary-like networks, these structures were not perfused and lacked defined inlet and outlet connections. Approaches for generating perfusable vascular compartments include bioprinting, sacrificial-channel fabrication, and microfluidic strategies in which endothelialized conduits are incorporated into dermal matrices [75]. More recently, human induced-pluripotent-stem-cell-derived vascular organoids have been integrated into skin models to generate complex vascular structures, and microfluidic studies have demonstrated that vascular organoids can functionally connect with external endothelial networks and support perfusion [76,77]. Coupling prevascularized HSEs with vascular organoids or a perfusable endothelial interface may therefore allow self-organized capillary-like networks to connect with an external circulation.

The use of HUVECs represents an additional limitation. Although they provide a reproducible and experimentally tractable endothelial-cell source, HUVECs may not fully reproduce the tissue-specific phenotype and functional properties of adult dermal microvascular endothelial cells. The model also lacks several components of native skin, including melanocytes, peripheral nerves, hair follicles, glands, and vascular support cells such as pericytes. Future studies will evaluate donor-to-donor variability, determine whether tissue stability can be maintained beyond six weeks, assess endothelial barrier and flow responses, and validate the platform using a broader range of pathogens, chemical exposures, and therapeutic interventions.

In conclusion, HSEs capture key structural and functional features of human skin, including epidermal stratification, development of a restrictive permeability barrier, stromal cellular complexity, endothelial self-organization, and maintenance of a p63-positive basal keratinocyte compartment during extended culture. The model also supports non-destructive longitudinal imaging and exhibits epithelial pathology and transcriptional responses consistent with established features of cowpox and orthopoxvirus infection. Collectively, these findings support the use of HSEs as a long-term in vitro platform for investigating tissue maturation, epidermal–stromal interactions, cutaneous infection, and injury responses. Incorporation of tissue-specific vascular cells, immune populations, additional skin cell types, and perfusion could further enhance the model’s physiological fidelity and translational utility.

## Data Availability

All data are available upon reasonable request. Requests for further information and resources should be directed to and will be fulfilled by the lead contact, P.M., at patrick.mcnutt@wfusm.edu.

## Supplementary Materials

The following supporting information can be downloaded at: https://www.mdpi.com/article/10.3390/jfb17080374/s1, Figure S1: Histology of HSEs cultured under different media compositions; Figure S2: SDS treatment destroys the epidermal barrier integrity of HSEs; Figure S3: Histology and collagen production in long-term cultured HSEs; Figure S4: HSEs are not susceptible to cowpox virus infection by topical administration; Video S1: Capillary-like network in HSEs.

## Abbreviations

The following abbreviations are used in this manuscript:

3D	Three-dimensional
ALI	Air–liquid interface
CK10 or KRT10	Cytokeratin 10
CK5 or KRT5	Cytokeratin 5
CLDN1	Claudin-1
COL1A1	Collagen type I alpha 1 chain
COL7A1	Collagen type VII alpha 1 chain
DAVID	Database for Annotation, Visualization, and Integrated Discovery
DEJ	Dermo-epidermal junction
FGM	Fibroblast growth medium
hpi	Hours post-infection
HSEs	Human skin equivalents
IFNs	Interferons
IPA	Ingenuity pathway analysis
IRF	Interferon regulatory factor
KSFM	Keratinocyte serum-free medium
LEP	Leptin
LOR	Loricrin
MOI	Multiplicity of infection
OCT	Optical coherence tomography
PGM	Preadipocyte growth medium
PLIN4	Perilipin-4
PPARG	Peroxisome proliferator-activated receptor gamma
TMM	Trimmed mean of M-values
